# Non-Newtonian Endothelial Shear Stress Simulation: Does It Matter?

**DOI:** 10.3389/fcvm.2022.835270

**Published:** 2022-04-14

**Authors:** Vikas Thondapu, Daisuke Shishikura, Jouke Dijkstra, Shuang J. Zhu, Eve Revalor, Patrick W. Serruys, William J. van Gaal, Eric K. W. Poon, Andrew Ooi, Peter Barlis

**Affiliations:** ^1^Department of Medicine, Faculty of Medicine, Melbourne Medical School, Dentistry and Health Sciences, University of Melbourne, Parkville, VIC, Australia; ^2^Department of Mechanical Engineering, Melbourne School of Engineering, University of Melbourne, Parkville, VIC, Australia; ^3^Department of Radiology and Biomedical Imaging, Yale University School of Medicine, New Haven, CT, United States; ^4^Department of Cardiology, Osaka Medical and Pharmaceutical University, Osaka, Japan; ^5^Department of Radiology, Division of Image Processing, Leiden University Medical Center, Leiden, Netherlands; ^6^Department of Biomedical Engineering, Melbourne School of Engineering, University of Melbourne, Parkville, VIC, Australia; ^7^Department of Cardiology, National University of Ireland Galway (NUIG), Galway, Ireland; ^8^National Heart and Lung Institute, Imperial College London, London, United Kingdom; ^9^Department of Cardiology, Northern Hospital, Epping, NSW, Australia

**Keywords:** computational fluid dynamics – CFD, non-Newtonian, rheology, viscosity, optical coherence tomography, shear stress (fluid)

## Abstract

Patient-specific coronary endothelial shear stress (ESS) calculations using Newtonian and non-Newtonian rheological models were performed to assess whether the common assumption of Newtonian blood behavior offers similar results to a more realistic but computationally expensive non-Newtonian model. 16 coronary arteries (from 16 patients) were reconstructed from optical coherence tomographic (OCT) imaging. Pulsatile CFD simulations using Newtonian and the Quemada non-Newtonian model were performed. Endothelial shear stress (ESS) and other indices were compared. Exploratory indices including local blood viscosity (LBV) were calculated from non-Newtonian simulation data. Compared to the Newtonian results, the non-Newtonian model estimates significantly higher time-averaged ESS (1.69 (IQR 1.36)Pa versus 1.28 (1.16)Pa, *p* < 0.001) and ESS gradient (0.90 (1.20)Pa/mm versus 0.74 (1.03)Pa/mm, *p* < 0.001) throughout the cardiac cycle, under-estimating the low ESS (<1Pa) area (37.20 ± 13.57% versus 50.43 ± 14.16%, 95% CI 11.28–15.18, *p* < 0.001). Similar results were also found in the idealized artery simulations with non-Newtonian median ESS being higher than the Newtonian median ESS (healthy segments: 0.8238Pa versus 0.6618Pa, *p* < 0.001 proximal; 0.8179Pa versus 0.6610Pa, *p* < 0.001 distal; stenotic segments: 0.8196Pa versus 0.6611Pa, *p* < 0.001 proximal; 0.2546Pa versus 0.2245Pa, *p* < 0.001 distal) On average, the non-Newtonian model has a LBV of 1.45 times above the Newtonian model with an average peak LBV of 40-fold. Non-Newtonian blood model estimates higher quantitative ESS values than the Newtonian model. Incorporation of non-Newtonian blood behavior may improve the accuracy of ESS measurements. The non-Newtonian model also allows calculation of exploratory viscosity-based hemodynamic indices, such as local blood viscosity, which may offer additional information to detect underlying atherosclerosis.

## Introduction

Fundamentally, computational fluid dynamics (CFD) is based on the idea that, given certain assumptions, the mechanics of fluid motion can be accurately described by physical principles and mathematical equations. The computational solution of these equations allows the determination of various hemodynamic indices such as blood velocity and pressure throughout the artery, from which other parameters such as endothelial shear stress (ESS) can be further derived. With continued advances, the underlying computational methods are frequently re-evaluated to optimize the shifting balance between accuracy and complexity. One such computational model concerns the variable viscosity of blood at high and low shear rates.

**FIGURE 1:**
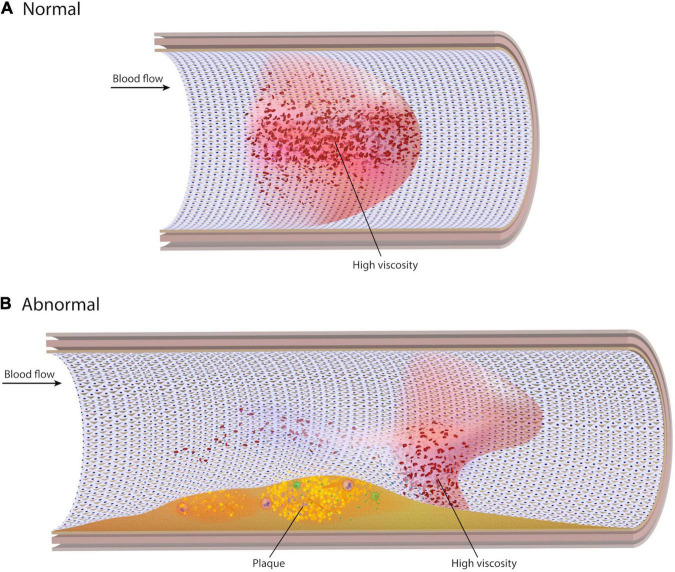
Normal and abnormal viscosity profiles. **(A)** In a straight unobstructed artery, blood velocity takes on a parabolic profile with low shear rate (high viscosity) in the centre of the artery and low viscosity at the wall. **(B)** In the presence of stenoses, curvatures, or bifurcations, the blood velocity profile becomes distorted. Localized regions of low shear rate can develop at the wall, resulting in high viscosity near the endothelial surface.

Earlier studies suggest that for laminar flow in medium to large arteries, blood may be assumed a Newtonian fluid with a constant viscosity independent of shear rate (1–5). However, due partly to its dual solid and liquid phases, blood exhibits non-Newtonian behaviors (6). This includes properties such as shear-thinning, the apparent thinning of blood at high shear rates and thickening at low shear rates. While the Newtonian assumption is generally acceptable in healthy straight segments of larger arteries, it may not be as accurate as non-Newtonian rheological models in the setting of complex flow patterns (7, 8). As artery anatomy changes, high fluctuations in local shear rate are significant enough that the non-Newtonian behavior of blood may emerge (Figure 1). In these near-wall regions, local blood viscosity (LBV) is expected to change from location to location and from instant to instant over the cardiac cycle. However, because the Newtonian model assumes constant viscosity, changes in LBV are not detected. The non-Newtonian model encompasses the variable viscosity of blood and thus provides this information. Our underlying hypothesis is that, if non-Newtonian behavior is negligible in coronary arteries, the two models should present nearly identical results. Divergent results, however, would suggest otherwise.

## Materials and Methods

### Study Design and Patient Selection

This study compares blood flow characteristics generated by CFD analysis in patient-specific coronary arteries using Newtonian and non-Newtonian blood models under pulsatile flow. Patients were retrospectively selected from a previous multicentre randomized clinical trial (NCT01776567). Inclusion criteria for the current study were the presence of an unstented, non-obstructive (diameter stenosis <50%) non-culprit lesion in the culprit vessel. Major exclusion criteria were ST-elevation myocardial infarction within the preceding 48 h, left ventricular ejection fraction <25%, and bifurcation lesions.

### Three-Dimensional Reconstruction

Patient-specific 3D arterial models were reconstructed through the fusion of OCT and angiography as previously described (9). The arterial centreline was extracted from two end-diastolic angiographic images with a >25° difference in viewing angles (QAngio XA 3D, Medis Specials Bv, Netherlands). Side branches outside the region of interest were used to co-register OCT images with angiograms. OCT lumen contours were semi-automatically detected and manually corrected as necessary (QCU-CMS, Leiden University Medical Center, Netherlands). OCT contours were placed onto the angiographic centreline (MATLAB R2017b, Mathworks Inc., Natick, MA, United States) and the vessel surface was generated (Solidworks, Dassault Systèmes, Velizy, France). Vessel volume was discretised into tetrahedral elements with an average mesh size of approximately 1 to 2 million depending on the geometric complexity of individual patient-specific arteries (Pointwise v18.2R2). All discretised models included a graduated 10-prism boundary layer to further enhance the resolution of flow phenomena near the arterial wall.

**FIGURE 2:**
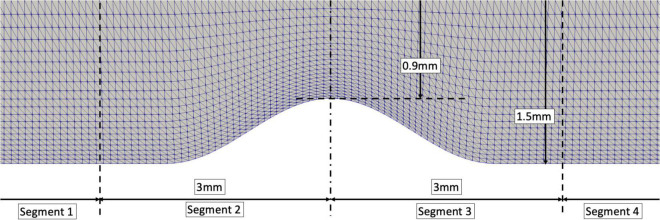
An idealized stenotic model with an artery’s diameter of 3mm and 40% DS. Segment 1: at least 1 diameter from the proximal stenotic segment; Segment 2: 1 diameter from the proximal shoulder of the stenosis to minimal lumen diameter; Segment 3: minimal lumen diameter to 1 diameter distal to the stenosis shoulder; Segment 4: remaining distal segment.

Given the uniqueness and complexity of an individual patient’s coronary arteries, it is difficult to isolate the specific geometric features impacting non-Newtonian blood rheology. Therefore, an idealized 2D model of intermediate stenosis was virtually created to assess the generalizability of the patient-specific results. The 2D model had a 3 mm diameter with diameter stenosis (DS) of 40%. The stenosis anatomy was assumed by the following mathematical equation:


$$D=\frac{1}{2}\,\left[D_{m\,i\,n}+\left(D-D_{m\,i\,n}\right)\,{\left(\sin\left(\pi \,x\right)\right)}^{2}\right]$$

where, *D* is the artery’s diameter, *D*_*min*_=DS×*D* is the minimum lumen diameter (MLD) and *x* is the longitudinal location of the stenotic segment with *x* = 0 at the MLD. The lesion length was assumed 3mm (i.e., *x* ranges from −1.5 to 1.5 mm). The idealized geometry was divided into 4 segments (Figure 2). Separating the proximal and distal stenotic geometries allows inspection of common flow phenomena – “favorable” and “adverse pressure gradient” – which are responsible for abnormal flow patterns such as flow separation and reversal.

### Computational Fluid Dynamics Simulation

Computational fluid dynamics analysis was accomplished through the direct solution of the Navier-Stokes equations describing fluid motion. OpenFOAM, a finite-volume CFD solver, was run on the Magnus supercomputer, consisting of 35,712 Intel Xeon E5-2690V3 “Haswell” processors (Pawsey Supercomputing Centre, Perth, WA, Australia). A time-varying parabolic velocity profile with a mean bulk velocity calculated from the patient-specific TIMI frame count as previously described was applied at the inlet (10). The arterial wall was considered rigid with a no-slip boundary and a non-specific distal vascular resistance was applied at the outlet. A resistance boundary condition assumes linear dependence between the pressure and flow rate at each outlet. It is analogous to a constant pressure boundary condition and is suitable for unsteady simulation with only single outlet (11).

For the Newtonian simulations, blood’s constant dynamic viscosity (μ_Newtonian_) was assumed 0.0035Pa⋅s. Blood density was considered 1,060kg/m^3^ and haematocrit 45%. Simulations were run for 3 cardiac cycles to ensure convergence of results, and all results presented are only from the final cycle. CFD results were post-processed to extract instantaneous and time-averaged hemodynamic indices where appropriate. ESS was calculated as described in Chen et al. (12). ESS gradient (ESSG) was calculated as the spatial gradient of ESS, representing the rate of change in ESS between adjacent spatial points in a local coordinate system (*x*′, *y*′, *z*′):


$$\text{ESSG}=\nabla \text{ESSG}=\left[\begin{array}{ccc} \frac{\partial {\text{ESS}}_{x^{\prime }}}{\partial x^{\prime }} & \frac{\partial {\text{ESS}}_{x^{\prime }}}{\partial y^{\prime }} & \frac{\partial {\text{ESS}}_{x^{\prime }}}{\partial z}\\ \frac{\partial {\text{ESS}}_{y^{\prime }}}{\partial x^{\prime }} & \frac{\partial {\text{ESS}}_{y^{\prime }}}{\partial y^{\prime }} & \frac{\partial {\text{ESS}}_{y^{\prime }}}{\partial z^{\prime }}\\ \frac{\partial {\text{ESS}}_{z^{\prime }}}{\partial x^{\prime }} & \frac{\partial {\text{ESS}}_{z^{\prime }}}{\partial y^{\prime }} & \frac{\partial {\text{ESS}}_{z^{\prime }}}{\partial z^{\prime }} \end{array}\right]$$

Since ESS represents the tangential force acting on the surface, all normal components (*z*′) of the tensor are irrelevant to the ESSG calculation. Removing all irrelevant tensor components in the *z*′ direction, the ESSG was simplified to:


$$\text{ESSG}=\left[\begin{array}{cc} \frac{\partial {\text{ESS}}_{x^{\prime }}}{\partial x^{\prime }} & \frac{\partial {\text{ESS}}_{x^{\prime }}}{\partial y^{\prime }}\\ \frac{\partial {\text{ESS}}_{y^{\prime }}}{\partial x^{\prime }} & \frac{\partial {\text{ESS}}_{y^{\prime }}}{\partial y^{\prime }} \end{array}\right]$$

and the ESSG magnitude is written as the diagonal components of the above matrix. Oscillatory shear index (OSI),


$$\text{OSI}=0.5\times \left(1-\frac{\left|\int_{0}^{t}\vec{\text{ESS}}\,dt\right|}{\int_{0}^{t}\left|\vec{\text{ESS}}\right|\,dt}\right),$$

indicates the degree of fluctuation in the direction of ESS vectors over the cardiac cycle. It is effectively an index of blood flow recirculation in a pulsating flow environment (13). Hemodynamic variables were calculated at 64 discrete timepoints per cardiac cycle (see Supplementary Table 1 for sensitivity analysis).

Similar procedures were employed in the non-Newtonian simulations except blood was modeled by the Quemada constitutive equation to capture the local variations in blood viscosity (μ_non–Newtonian_) (14). Local blood viscosity (LBV) was expressed as a ratio of non-Newtonian viscosity to the Newtonian constant viscosity model (μ_non–Newtonian_ /μ_Newtonian_) for each tetrahedral element (15). In other words, LBV is a quantitative measure of the impact of non-Newtonian flow relative to that assumed by the Newtonian model. A value of 1 indicates no effect and any values >1 demonstrate the proportional influence of non-Newtonian properties locally in the bloodstream.

### Statistical Analysis

Two simulations were carried out for each patient, one using the Newtonian model and the second using a non-Newtonian model. Since the reconstructed arterial models and computational meshes were identical for the Newtonian and non-Newtonian simulations for each patient, a rigorous point-by-point comparison of all vessel nodes was possible. Due to the paired nature of observations, the only variable within each patient was the choice of rheological model. Thus, paired comparisons of the Newtonian and non-Newtonian results were performed, as described below.

Time-averaged ESS and ESSG were calculated for each case. Instantaneous ESS was evaluated at every spatial point on the arterial wall at every step in the cardiac cycle. The maximum ESS at that spatial point was identified. To eliminate the skewing effect of extremely high and low ESS values inherently present in complex geometries, ESS was normalized using the maximum ESS value at every spatial point, yielding a value between 0 and 1. At each time step, a point-by-point comparison of normalized ESS yielded the normalized difference between the Newtonian and non-Newtonian models. The same methods were used to analyze ESSG whereas, by definition, OSI describes the general flow behavior over a cardiac cycle. Similar analyses were also carried out on the four segments of the idealized artery geometry.

Categorical variables are presented as counts and percentages, while continuous variables are presented as a mean ± standard deviation and non-parametric variables in median (interquartile range [IQR]). Because of the paired nature of the simulations within each patient, the only variable was the choice of rheological model, therefore paired *t*-test or Wilcoxon sign rank test for paired observations (as appropriate) were used. To avoid statistical dependence and to decorrelate data in the point-by-point comparison, bootstrap resampling with replacement was used to randomly select 1.5% of all mesh points and the above-mentioned statistical tests were performed to compare the results of Newtonian and non-Newtonian simulations. This process was repeated 10,000 times for comparison of ESS, ESSG, and OSI. All tests were two-tailed with an α-level of 0.05 to indicate statistical significance. Statistical analysis was performed in R statistical software (R Foundation for Statistical Computing, Vienna, Austria).

## Results

### Wall-Based Indices: Endothelial Shear Stress, ESS Gradient, and Oscillatory Shear Index

**FIGURE 3:**
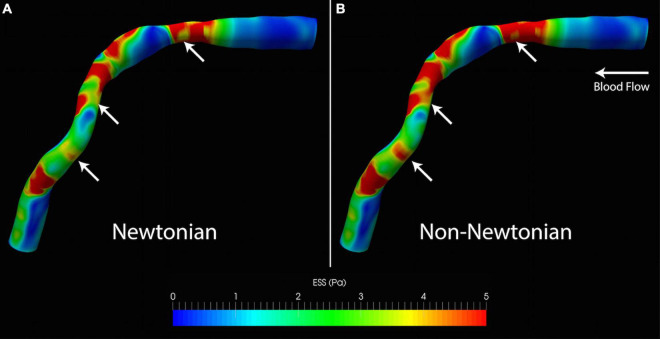
Qualitative differences in ESS from **(A)** Newtonian and **(B)** non-Newtonian models. Although the distribution of high and low ESS is similar, the non-Newtonian model predicts higher ESS throughout the artery. This is visually most apparent from the larger areas exposed to high ESS (white arrows). ESS, endothelial shear stress.

Patient characteristics are shown in Table 1. Qualitative comparison of time-averaged ESS between the Newtonian and non-Newtonian simulations demonstrates broad similarity in its range and distribution for all cases (Figure 3). However, notable differences are found at the stenosis and curved segments (white arrows). By quantitative comparison, time-averaged ESS in the non-Newtonian simulations was significantly higher (1.69 [1.36]Pa versus 1.28 [1.16]Pa, *p* < 0.001), translating to a mean normalized percent difference of 21.72% over the entire cardiac cycle. Time-averaged ESSG in the non-Newtonian simulations was also significantly higher than the Newtonian model (1.65 ± 0.92Pa/mm versus 1.37 ± 0.78Pa/mm, 95% CI 0.20–0.37.16, *p* < 0.001). However, OSI was not significantly different between the models (0.0302 ± 0.035 versus 0.0294 ± 0.039, 95% CI 0.0059–0.0075, *p* = 0.81) (Table 2). The results of the analysis based on bootstrap resampling showed that the 95% confidence intervals of the mean and standard deviation/median and IQR are fully consistent with the analyses conducted on the raw data (Supplementary Table 2).

**TABLE 1:** Patient characteristics.

	*N* = 16
Age (years)	64.5
Male	13 (81.3)
Diabetes	3 (18.8)
Hypertension	8 (50)
Dyslipidemia	13 (81.3)
Current smoker	2 (12.5)
Former smoker	10 (62.5)
Previous myocardial infarction	5 (31.1)
Previous coronary artery bypass graft	0
Previous percutaneous coronary intervention	4 (25)
**Vessel**	
Left anterior descending artery	10 (62.5)
Right coronary artery	2 (12.5)
Left circumflex artery	4 (25)
Statin	14 (87.5)
**Presentation**	
Stable	9 (56.3)
Unstable	2 (12.5)
Non-ST elevation myocardial infarction	5 (31.3)
**Simulation variables**	
Inlet flow (mL/s)	0.83 ± 0.44
Length of region of interest (mm)	13.40 ± 4.21

**TABLE 2:** ESS, ESSG, and OSI between rheological models.

	Non-Newtonian	Newtonian	*p*-value
ESS (Pa), median (IQR)	1.69 (1.36)	1.28 (1.16)	<0.001
ESSG (Pa/mm), median (IQR)	0.90 (1.20)	0.74 (1.03)	<0.001
OSI, mean ± SD	0.0302 ± 0.035	0.0294 ± 0.039	0.81

*ESS, endothelial shear stress; ESSG, endothelial shear stress gradient; IQR, interquartile range; OSI, oscillatory shear index; SD, standard deviation.*

**FIGURE 4:**
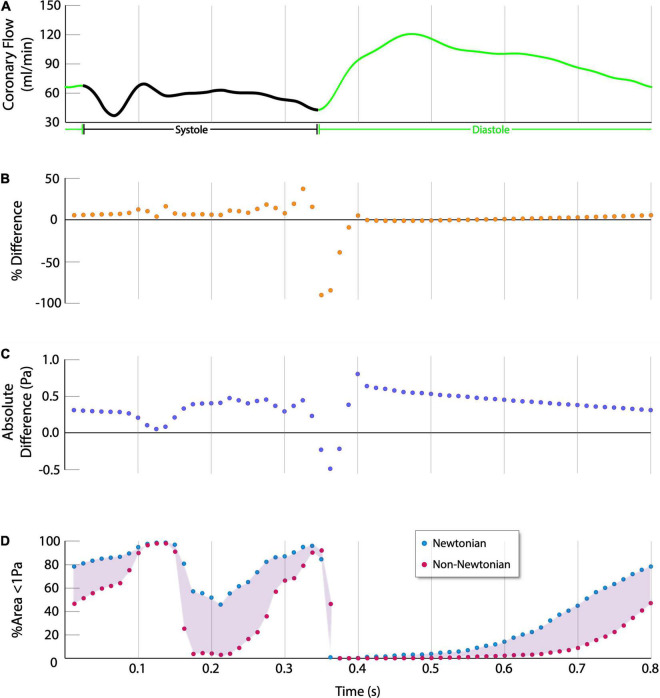
Quantitative difference in ESS between Newtonian and non-Newtonian models (Single Representative Case). **(A)** Coronary flow rate, systole is indicated in black, diastole is indicated in green. **(B,C)** The % difference and absolute difference are calculated as (non-Newtonian ESS – Newtonian ESS), thus positive values indicate non-Newtonian results were higher whereas negative values indicate higher Newtonian results. The non-Newtonian results show consistently higher percent normalized difference and absolute difference in ESS over the cardiac cycle, except during the momentary transition between systole and diastole at approximately 0.35s. **(D)** Newtonian simulations predict more of the vessel is exposed to atherogenic levels of ESS over the cardiac cycle.

The absolute and normalized percent difference in instantaneous ESS and ESSG relate inversely with coronary flow rate. At the higher coronary blood flow rates associated with diastole, the difference between Newtonian and non-Newtonian simulations approaches zero, with a minimum difference in absolute ESS of 0.035 ± 0.036Pa (Figure 4). However, the difference increases at low and decelerating flow rates that characterize systole, with a maximum difference in absolute ESS of 1.05 ± 0.42Pa (Figures 4B,C).

Although the time-averaged results show that the non-Newtonian simulations estimate higher ESS and ESSG over the cardiac cycle, during the momentary transition between end-systole and early diastole, at approximately 0.35s into the cardiac cycle, the Newtonian ESS results are higher than the non-Newtonian results by 0.89 ± 0.52Pa (*p* < 0.001), or 946.97 ± 898.72% (*p* < 0.001). However, this is transient, and the non-Newtonian results again become higher immediately thereafter (Figures 4B,C). The non-Newtonian model predicts the highest ESS relative to the Newtonian model during early diastole, as coronary flow is increasing rapidly. However, as the rate of rise slows in mid-diastole (at approximately 0.4s), non-Newtonian ESS is higher by 0.96 ± 0.25Pa (*p* < 0.001), or 58.13 ± 22.12% (*p* < 0.001).

The implications of these differences are most apparent by comparing vessel areas predicted to be exposed to low ESS (<1Pa), a generally accepted threshold for stimulating pro-atherogenic processes (16, 17). The Newtonian model predicts significantly greater vessel area exposure to low ESS than the non-Newtonian model (50.43 ± 14.16% versus 37.20 ± 13.57%, 95% CI 11.28–15.18%, *p* < 0.001) (Figure 4D).

Results from the idealized arterial geometries are consistent with the 3D patient-specific results (Table 3). ESS was higher in the non-Newtonian simulation in both proximal and distal healthy segments (0.8238Pa versus 0.6618Pa, *p* < 0.001 and 0.8179Pa versus 0.6610Pa, *p* < 0.001, respectively); in proximal and distal stenotic segments (0.8196Pa versus 0.6611Pa, *p* < 0.001 and 0.2546Pa versus 0.2245Pa, *p* < 0.001, respectively). Both non-Newtonian and Newtonian simulations display similar IQR ESS, except for the distal stenotic segment where non-Newtonian simulation demonstrates a significantly higher IQR than Newtonian (0.2199Pa versus 0.08843Pa, *p* < 0.001). IQR is a measure of the data dispersion (18). In other words, IQR indicates the range of ESS within the region of interest. High IQR indicates wider spread of the ESS values and hence larger variation of ESS from the median, a condition that can be found with increasing chaotic blood flow due to vortices and flow oscillations.

**TABLE 3:** ESS distribution between rheology models in an idealized artery (as depicted in Figure 1).

	Newtonian	Non-Newtonian	*p*-value
Segment 1, median ESS (IQR)	0.6618 (0.0000)	0.8238 (0.0015)	<0.001
Segment 2, median ESS (IQR)	0.6611 (0.2441)	0.8196 (0.2288)	<0.001
Segment 3, median ESS (IQR)	0.2245 (0.0884)	0.2546 (0.2199)	<0.001
Segment 4, median ESS (IQR)	0.6610 (0.0007)	0.8179 (0.0059)	<0.001

*ESS, endothelial shear stress; IQR, interquartile range.*

### Local Blood Viscosity

**FIGURE 5:**
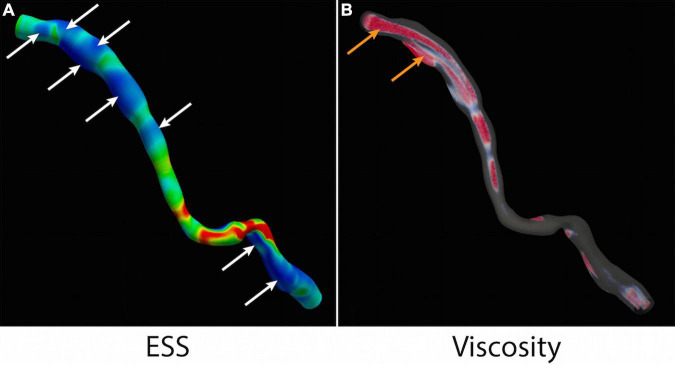
ESS and local blood viscosity. **(A)** Multiple areas of low ESS (<1Pa) are identified (white arrows). **(B)** Non-Newtonian simulation detects regions of high LBV (>1.45) in the centre of the vessel and at the wall (orange arrows). ESS, endothelial shear stress; LBV, local blood viscosity.

Like ESS, there is high spatial and temporal heterogeneity in blood viscosity over the cardiac cycle. Localized volumetric regions of high blood viscosity are observed in every case, including at the centre and walls of the artery. Across all cases, time-averaged viscosity was 1.45-fold higher than that assumed by the Newtonian model (95% CI 1.43–1.49, *p* < 0.001) (Figure 5). Some vessel regions are marked by an average 41.5-fold increase in maximum viscosity compared to the Newtonian model (95% CI 30.1–53.0, *p* < 0.001). In one case, peak viscosity was more than 70 times higher. The peak viscosity invariably occurs during a nadir in coronary flow – either during peak systole or the transition between end systole and early diastole.

## Discussion

This study demonstrates: (1) the non-Newtonian model predicts significantly higher ESS and ESSG than the Newtonian model; (2) the Newtonian model shows significantly greater vessel areas exposed to atherogenic low ESS; (3) OSI is not significantly different between the models; and (4) the non-Newtonian model identifies regions of high LBV up to 70-fold higher than that assumed by the Newtonian model.

Local blood flow disturbances are a potent stimulus of endothelial dysfunction and biological processes underlying atherosclerosis (16, 19, 20). ESS disturbances *in vivo* have been correlated with changes in plaque composition (21, 22), morphology (23), and vessel remodeling (21, 22). Further, low ESS is independently associated with requiring future intervention or even future clinical events (24, 25). In these studies, many vessel areas were predicted to be exposed to low ESS; of lesions that caused future events, most or all were in previously low ESS areas; however, the vast majority of lesions within low ESS areas did not progress to cause events. Previous studies indicate that, while low ESS has high sensitivity to detect future events at a patient level, it has low specificity and positive predictive value (PPV) (24, 25). Furthermore, no studies have associated specific plaques exposed to low ESS as the culprit lesion of later events.

Although blood is a non-Newtonian fluid, all these studies used the Newtonian model of blood behavior. Since coronary blood flow is assumed to have shear rates above 100–200s^–1^, such an assumption has long been valid. We investigated the effects of non-Newtonian blood rheology in a series of coronary arteries reconstructed from high-resolution imaging to test whether the Newtonian and non-Newtonian models present results within a small margin of computational error. In other words, if the shear rate was indeed high enough that blood can be assumed a Newtonian fluid, the two models should predict quantitatively similar results. However, the results of this study show that the Newtonian and non-Newtonian models consistently estimate different ESS values throughout the cardiac cycle, suggesting that there are indeed multiple factors, including the instantaneous pulsatile blood flow velocity and local geometric variations, influencing when and where the non-Newtonian behaviors of blood become apparent.

### Pulsatile Flow Factor

Unlike Newtonian CFD simulations where the blood viscosity stays constant throughout the cardiac cycle, blood viscosity constantly changes from moment to moment due to the pulsatile nature of blood flow. Indeed, in a Newtonian simulation, the sudden increase in coronary blood flow rate at early diastole is usually accompanied by a very rapid increase in ESS untamed by the constant viscous forces and vice versa. However, the non-Newtonian fluid responds differently to temporal changes in flow rate and hence leads to a very dynamic pattern of the discrepancy between the Newtonian and non-Newtonian results. While non-Newtonian simulations, in general, predict a higher ESS, there is a time point between end-systole and early diastole where the non-Newtonian model momentarily predicts significantly lower ESS than the Newtonian model. Coincidentally, the difference between the two models is most remarkable during this transition phase. While it can be easily speculated that the rapidly changing blood flow conditions play a role, the underlying haemodynamics are far more complicated. One must consider the heart rate, the historical effects of LBV, and its impact on the local flow environment.

Nevertheless, these findings are consistent with many previous fundamental studies showing that non-Newtonian simulations predict higher ESS (26–31). For instance, non-Newtonian flow decreases the area of low ESS in both straight and bent arterial segments, with the largest difference occurring in the straight rather than the bent segment (32). On the other hand, while blood viscosity affects the magnitude of ESS when the flow is disturbed, it does not affect the spatial and temporal distribution of the ESS (33, 34). Our results demonstrate that those results extend to patient-specific coronary arteries. It should be noted that while this difference was also observed for ESSG, there was no significant difference in predicted OSI values.

Reassuringly, ESS calculated by the non-Newtonian model is, on average, 0.44Pa or 21% higher than the Newtonian model in the 3D patient-specific data and 0.63Pa or 27% in the idealized 2D results. While the absolute value of the difference between Newtonian and non-Newtonian models is low, this ultimately means that the Newtonian model estimates a significantly higher percentage of the vessel area exposed to ESS <1Pa (50.43% versus 37.20%, *p* < 0.001). This could have significant repercussions in the context of earlier clinical CFD studies showing low specificity and PPV of low ESS. Despite the estimated differences in ESS and ESSG, the clinical significance of such fluctuations within a short time is unclear. However, it is hypothesized that the non-Newtonian model, as a more accurate reflection of actual blood behavior, may ultimately offer higher specificity and PPV than Newtonian simulations.

### Geometric Factor

Arterial narrowing and widening are major factors in local variations of blood rheology and flow dynamics. Blood flow accelerates as the artery narrows. In haemodynamics, this segment is termed the “favorable pressure gradient” segment. In contrast, “adverse pressure gradient” refers to flow deceleration as the artery widens. The impact of “favorable” and “adverse pressure gradients” can be isolated by carrying out the Newtonian and non-Newtonian simulations in an idealized arterial geometry where the arterial flow will undergo clearly defined favorable (proximal stenotic segment) and adverse (distal stenotic segment) pressure gradients. Our idealized artery results show that the non-Newtonian model continues to display a higher median ESS value compared to the Newtonian model in all segments. However, it is the significantly lower IQR at the distal stenotic segment marked by an adverse pressure gradient in the Newtonian model that is of particular interest. While low IQR reflects lower ESS oscillation and high IQR indicates larger fluctuations in ESS values, it is unclear whether a higher IQR signifies an increase in turbulent activities or vice versa. Nonetheless, regions with adverse pressure gradients have demonstrated an increased likelihood of flow reversal (8). Clinically, flow reversal and abnormal ESS represent a location for plaque development and potentially a nidus for thrombotic events (35–37). This remarkable difference in Newtonian and non-Newtonian models in regions of adverse pressure gradients might lead to different conclusions and hence warrants further objective analyses.

### The Need for a Better Reflection of True Blood Rheology

Non-Newtonian models offer other potential advantages. Although ESS, ESSG, OSI, and other wall-based metrics consider the mechanical effect of blood acting on the vessel wall, these indices inherently neglect the physiological response of whole blood. While low ESS and high OSI indicate that blood may be recirculating and stagnating in these areas, these measures do not directly capture or describe flow phenomena within the blood itself.

Compared to the constant viscosity assumed by the Newtonian model, in non-Newtonian rheological models, blood viscosity is treated as a variable dependent on instantaneous local shear rate, allowing determination of viscosity within the entire fluid domain. In this study, the non-Newtonian model identified localized regions of peak LBV, on average, 40-fold higher than that assumed by the Newtonian model. The possibility of detecting localized regions of increased blood viscosity *in vivo* is intriguing given that blood is a complex fluid with clinically relevant behaviors such as thrombosis. Further, the pathophysiologic mechanisms underlying plaque development may involve the accumulation of cholesterol, pro-inflammatory cells, and humoral mediators in characteristic vessel regions, perhaps exacerbated by increased LBV, recirculation and stagnation in these areas.

In straight unobstructed vessels, high viscosity is expected in the center of a vessel where shear rate is low, and velocity is high (Figure 1). Conversely, blood viscosity at the wall is low since the shear rate at the wall is high. It is hypothesized that, despite high viscosity at the vessel centre, high velocity convects blood axially downstream, preventing significant erythrocyte aggregation or contact with the endothelial surface. However, low blood velocity and recirculation can develop at the distal inner bend of curvatures, the outer walls of bifurcations, and both proximal and distal to stenoses or stent struts. In such regions of disturbed flow, blood velocity and shear rate may decrease at the wall leading to pockets of high LBV near the endothelial surface, potentially facilitating processes leading to both progressive atherosclerosis and thrombosis (38, 39).

### Limitations

There are several limitations to the current study. First is a small study population retrospectively selected from a prior randomized clinical trial. However, in the context of patient-specific CFD studies quantitatively comparing rheological models, this is among the largest cohorts. As a result of retrospective patient selection, the cohort also skewed male due to the original study population characteristics (75% male in the original study). Second, we assumed a generic haematocrit of 45% based on standard reference ranges and a desire to limit confounding variables that might have been introduced by incorporating patient-specific values. However, because haematocrit is a determinant of blood viscosity, it is possible that the observed differences between the Newtonian and non-Newtonian models may also be influenced by changes in haematocrit. Future studies should incorporate patient-specific haematocrit to better assess this possibility. Third, this study does not investigate the effect of axial and secondary flow due to the presence of helical inflow, arterial curvature, bifurcation lesions which significantly affect local flow dynamics (40). It is expected that due to the increased complexity of flow in these settings, the differences between the Newtonian and non-Newtonian models would increase further. To facilitate investigating these effects, it is important to correlate LBV with other ESS-based descriptors and helicity indices (41, 42). In terms of bifurcation lesions, future studies should prospectively image-side branches with intravascular techniques if it is feasible and safe to do so. Fourth, as a technical study, changes in plaque composition were not evaluated. Further investigation of ESS derived by Newtonian and non-Newtonian models concerning plaque composition and change over time is necessary. Ultimately, if there is a significant difference in how the models correlate with atherosclerotic plaque, future studies investigating clinical endpoints may be worthwhile.

## Conclusion

Although blood is often assumed to be a Newtonian fluid by CFD simulations of the coronary arteries, this study demonstrates that non-Newtonian behaviors of blood are operational, yielding marked differences in calculated flow indices such as ESS and ESSG. Non-Newtonian simulations also allow the calculation of LBV and related indices, potentially presenting novel markers to detect plaques at risk for progression.

## Data Availability Statement

The CFD data supporting the conclusions of this article will be made available by the authors. Request for access to patient data (which are subjected to restriction due to the nature of personal healthcare information) can be made to the corresponding author.

## Ethics Statement

The studies involving human participants were reviewed and approved by St Vincent’s Ethics Committees (APPOSE HREC/12/SVH/31). The patients/participants provided their written informed consent to participate in this study.

## Author Contributions

VT designed the methodology, performed 3D reconstructions and CFD analysis and wrote the original manuscript, and reviewed and edited the manuscript. JD supported intravascular optical coherence tomography analysis. SZ contributed to the CFD modeling. DS, ER, and WG reviewed and edited the manuscript. PS provided expert opinion, review and edited the manuscript. EP co-designed the methodology and performed CFD analysis, co-supervised the project. AO contributed to CFD methods and co-supervised the project. PB collected all medical imaging data, conceptualized the project, supervised, and co-ordinated the project. All authors contributed to the article and approved the submitted version.

## Conflict of Interest

PS reports personal fees from Sino Medical Sciences Technology, Philips/Volcano, and Xeltis. The remaining authors declare that the research was conducted in the absence of any commercial or financial relationships that could be construed as a potential conflict of interest.

## Publisher’s Note

All claims expressed in this article are solely those of the authors and do not necessarily represent those of their affiliated organizations, or those of the publisher, the editors and the reviewers. Any product that may be evaluated in this article, or claim that may be made by its manufacturer, is not guaranteed or endorsed by the publisher.
