# Supplementary material for: Non-Newtonian Endothelial Shear Stress Simulation: Does It Matter?
Source: Front Cardiovasc Med. 2022 Apr 14;9:835270. doi: 10.3389/fcvm.2022.835270 (PMC9046559; doi:10.3389/fcvm.2022.835270)
Supplement: Supplementary file 1 [file Data_Sheet_1.PDF]

# *Supplementary Material*

## 1 Supplementary Tables

| Temporal Resolution | Median ESS | % difference | Median ESSG | % difference | Median OSI | % difference |
|---|---|---|---|---|---|---|
| **32 time-steps** | 0.7981 | -0.31% | 0.6375 | 2.79% | 0 | 0 |
| **64 time-steps** | 0.8006 | - | 0.6202 | - | 0 | - |
| **128 time-steps** | 0.8026 | 0.25% | 0.6169 | -0.53% | 0 | 0 |

**Supplementary Table 1.** Sensitivity analysis. ESS, ESSG, and OSI were calculated using 32, 64, and 128 time-steps. All solutions are within 5% of each other. As increasing from 64 to 128 time-step resulted in marginal difference, all time-averaged solutions were reported using the 64 time-steps studies.

| | Raw Data | Bootstrap Resampled Data (R=10000) | |
|---|---|---|---|
| | | 95% CI of Mean or Median | 95% CI of Standard Deviation or IQR |
| **Newtonian ESS, median (IQR)** | 1.28 (1.16) | 1.22-1.35 | 1.05-1.26 |
| **Non-Newtonian ESS, median (IQR)** | 1.69 (1.36) | 1.61-1.76 | 1.24-1.50 |
| **Newtonian ESSG, median (IQR)** | 0.74 (1.03) | 0.69-0.80 | 0.92-1.14 |
| **Non-Newtonian ESSG, median (IQR)** | 0.90 (1.20) | 0.84-0.97 | 1.08-1.33 |
| **Newtonian OSI, mean ± SD** | 0.0349 (0.0575) | 0.031-0.039 | 0.050-0.068 |
| **Non-Newtonian OSI, mean ± SD** | 0.0351 (0.0467) | 0.032-0.038 | 0.042-0.052 |

**Supplementary Table 2.** Analysis based on bootstrap resampling showed that the 95% confidence intervals of the mean and standard deviation/median and IQR against the raw data.
